# Two-Photon Absorbing Dendrimers and Their Properties—An Overview

**DOI:** 10.3390/ijms25063132

**Published:** 2024-03-08

**Authors:** Valérie Maraval, Anne-Marie Caminade

**Affiliations:** 1 Laboratoire de Chimie de Coordination (LCC) du CNRS, 205 Route de Narbonne, 31077 Toulouse Cedex 4, France; anne-marie.caminade@lcc-toulouse.fr; 2 LCC-CNRS, Université de Toulouse, CNRS, 31077 Toulouse, France

**Keywords:** dendrimer, two-photon absorption, optical power limitation, photopolymerization, light harvesting, photosensitizer, photodynamic therapy, bioimaging

## Abstract

This review describes the two-photon absorption properties of dendrimers, which are arborescent three-dimensional macromolecules differing from polymers by their perfectly defined structure. The two-photon absorption process is a third order non-linear optical property that is attractive because it can be used in a wide range of applications. In this review, dendrimers that were studied for their two-photon absorption properties are first described. Then, the use of dendritic TPA chromophores for light harvesting, photopolymerization, optical power limitation, cell imaging, singlet oxygen generation, and photodynamic therapy is described. This review thus proposes an overview of the properties and possible applications of two-photon absorbing dendrimers.

## 1. Introduction

After the theoretical prediction of the two-photon absorption (TPA) process by M. Göppert Mayer in 1931 [1], and the first experimental evidence of the phenomenon 30 years later [2], this third-order non-linear optical property has received considerable attention. Indeed, the TPA process is involved in a wide range of applications such as three-dimensional microfabrication [3], optical limitation [4], photodynamic therapy [5], or optical data storage [6]. Depending on the targeted application, TPA chromophores have to exhibit specific characteristics such as high fluorescence and/or solubility in aqueous media, for example. Many structure activity relationship studies were reported, and a large number of new organic dyes were designed and synthesized with the objective of reaching high TPA efficiencies [7]. The efficiency of a TPA chromophore is given by its cross-section value (*σ*TPA), expressed in Göppert-Mayer (1 GM = 10^−50^ cm^4^ s photon^−1^).

In the design of chromophores for two-photon absorption, dipolar structures D-π-A, in which a donor group D is separated from an acceptor group A by a π-conjugated bridge, were first investigated [8], followed by quadrupolar molecules D-π-D or A-π-A [9]. These studies evidenced that D-π-D or D-π-A-π-D chromophores are generally more efficient than A-π-A or A-π-D-π-A counterparts [10]. Later, octupolar branched molecules were investigated and were shown to be efficient TPA chromophores [11].

Dendrimers, macromolecules exhibiting a large number of functional groups at their surface, appeared as the natural next targets to investigate as TPA chromophores. Indeed, dendrimers are a special kind of perfectly defined hyperbranched polymers constructed stepwise from a multifunctional core [12]. Their synthesis is most of the time performed through a divergent method and involves the use of a branched monomer. The repetition of a two-step sequence of reactions allows the progressive growth of the branches of the dendrimer and an increase in the number of terminal functions. A new generation (Gn + 1) of the dendrimer is created each time the number of functional groups is multiplied, generally by two [13] (up to five in some cases) [14], thanks to the use of the branched monomer. Dendrimers are thus defined by their core, their branches, their generation (a new generation being created each time a branching point is introduced through the use of the branched monomer) and their peripheral (or surface) functions. In the last decades, dendrimers were reported to possess a large variety of properties, depending on their structure, size, and functionalization at the surface or core, and to open up prospects for many applications in the fields of nanomaterials, catalysis, or biology, for example [15].

In this review, dendrimers exhibiting two-photon absorption properties are first described. Then, the use of these dendritic TPA chromophores in various domains such as optical limitation, photopolymerization, light harvesting, bioimaging, singlet oxygen generation, or photodynamic therapy is presented. The originality of the proposed review resides in the fact that it is, to our knowledge, the first time that the dendritic TPA chromophores are presented depending on the different kind of properties and applications induced by the biphotonic excitation. The main objective is thus to illustrate the wide scope of applications that can be envisaged with dendritic TPA chromophores. 

## 2. Two-Photon Absorbing Dendrimers

**Figure 1:**
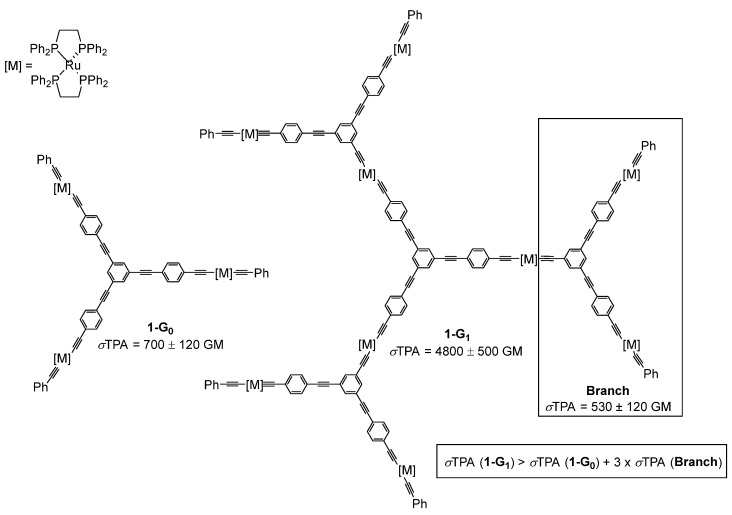
First example of TPA cross-section measurements of organometallic dendrimers.

The first report of a TPA cross-section of a dendrimer was published by M. G. Humphrey in 1999 [16]. This pioneer study was performed on organometallic alkynylruthenium dendrimers and showed that the TPA cross-section value measured for **1-G_1_** (*σ*TPA = 4800 ± 500 GM) was much higher than the value obtained for the smaller dendrimer **1-G_0_** (*σ*TPA = 700 ± 120 GM) (Figure 1). Interestingly, the value for **1-G_1_** was higher than the one calculated by the addition of the values measured for each constitutive motif of the molecule. This non-linear increase of the TPA cross-section values from **1-G_0_** to **1-G_1_** was the first observation of a dendritic effect, or multivalence effect, on the TPA property. We can speak of the dendritic effect when a function of a substituent grafted to the periphery of a dendrimer behaves differently depending on the size (or generation) of the dendrimer to which it is attached. Such dendritic effects, though not clearly explained, have been observed in different domains, such as for example in catalysis and the TPA properties [17].

**Figure 2:**
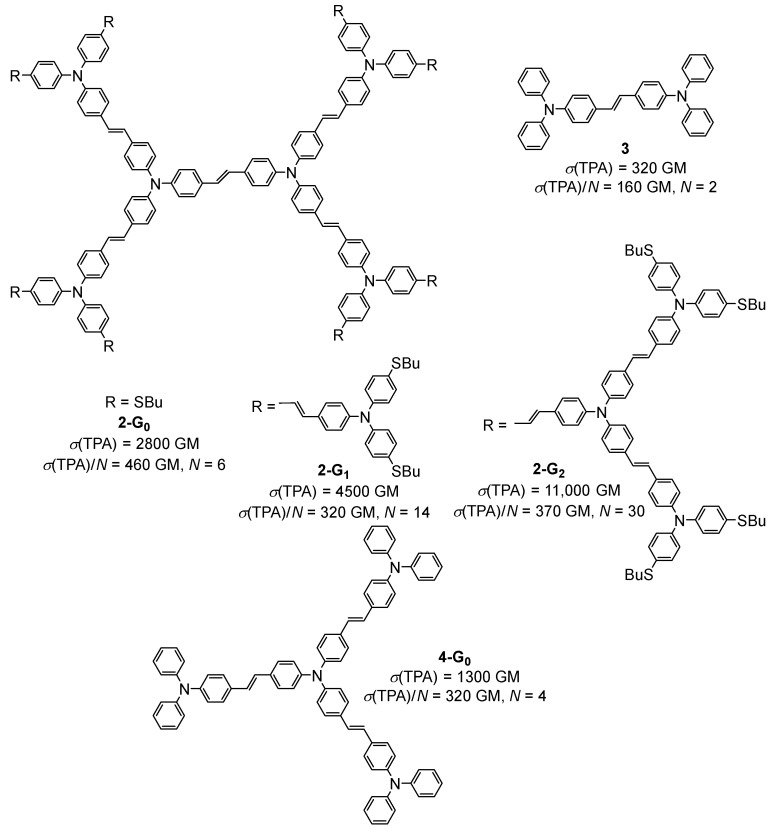
Structures and TPA cross-section values of bis-(diphenylamino)stilbene-based dendrimers.

In 2004, the TPA properties of the same kind of alkynylruthenium dendrimers bearing acceptor nitrophenyl groups at the periphery were investigated by the same authors [18]. No TPA process was observed with this nitro-substituted dendrimer upon irradiation at 800 nm. The combination of experimental data and theoretical calculations showed that at this wavelength, there is absorption saturation, and that the TPA process can only occur at a higher wavelength (around 1200 nm). These dendrimers were also shown to exhibit electrochemically induced switching of their NLO properties, in particular their nonlinear refraction and nonlinear absorption [19]. Later, the synthesis of a second generation of this type of alkynyl-metal dendrimer was reported, along with an investigation of the effect of the generation on nonlinearities [20]. Comparisons of the TPA properties relative to the number of ruthenium complexes, the molecular weight, or the number of delocalizable electrons all provided the same conclusions, i.e., that the NLO response increases with the generation, and that this increase is not only due to the increase of the molecular size, thus indicating a dendritic effect. In 2016, the same authors reported on the measurement of three- and four-photon absorption cross-section values for a ruthenium alkynyl dendrimer with nine ruthenium complexes in its structure. The *σ*(4PA) of 2100.10^−110^ cm^8^ s^3^, measured for this dendrimer upon irradiation at 1600 nm, was a record value at that time [21]. In 1999, the same year as the first report on the TPA properties of organometallic dendrimers [16], a preliminary evaluation of the TPA efficiency of the organic dendrimer **2-G_0_** based on bis-(diphenylamino)stilbene units was published [22]. These preliminary data were later confirmed and completed with measurements performed on the corresponding first- and second-generation dendrimers **2-G_1_** and **2-G_2_** (Figure 2) [23]. A cooperative effect was later demonstrated by comparison of the TPA efficiencies of **2-G_0_** with the monomer **3** and the related dendrimer **4-G_0_** constructed from a trifunctional core. 

In this study, the TPA cross-section values were expressed as a function of the number of chromophore units in the molecules, namely the number of triphenylamine moieties, thus evidencing that **2-G_0_** is the most efficient TPA chromophore of the series and that the *σ*(TPA)/N values of the three generations of dendrimers **2-G_n_** (n = 0–2) are almost similar. This latter observation was explained by a phenomenon of saturation when the dendrimers become larger [24].

Several theoretical studies were performed on this family of bis-diphenylamino)stilbene-based dendrimers of different generations and with different types of cores [25,26,27]. The TPA cross-section values were theoretically calculated, showing that these dendrimers exhibit very large TPA cross-sections and have a good transparency, making them promising materials for optical power limitation. Their TPA efficiency was calculated to increase linearly with the number of stilbene motifs in their structure, giving theoretical values up to 100,000 GM for **2-G_1_**, this theoretical value being largely overestimated as compared to the experimental value of 4500 GM.

**Figure 3:**
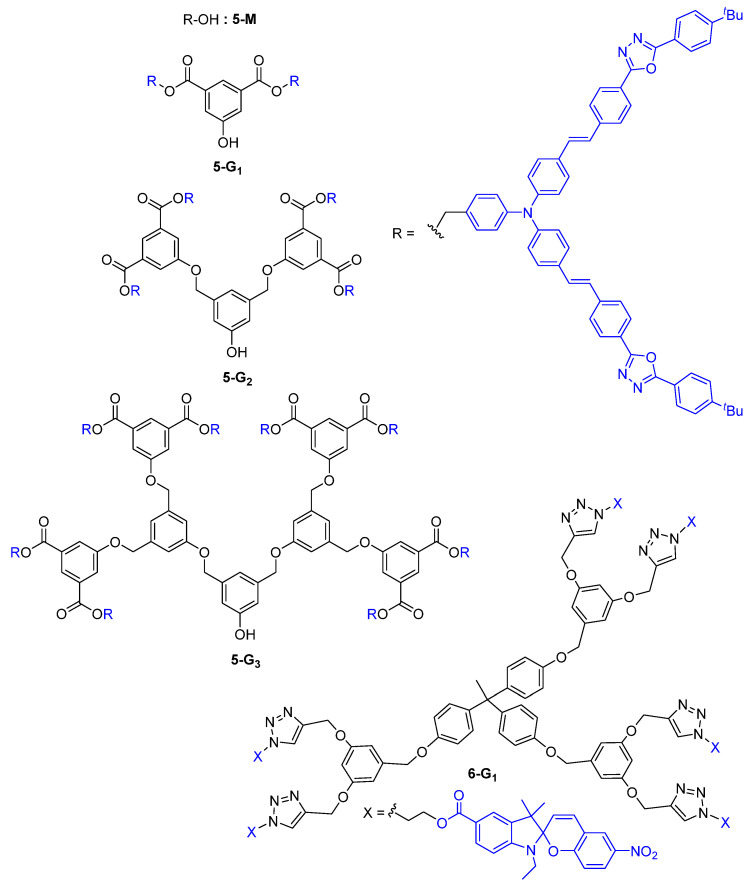
Non-conjugated dendrimers envisaged as TPA chromophores.

In 2000, one of the first reports on the use of dendrimers as TPA chromophores described the convergent synthesis of first-, second-, and third-generation dendrons **5-G_1_**, **5-G_2_**, and **5-G_3_** with a phenol core and the TPA chromophore **5-M**, embedding the bis-diphenylamino)stilbene motive in its structure at the periphery (Figure 3) [28].

The relative TPA cross-section of the monomer **5-M** and dendrons **5-G_n_** was found to double each time the number of chromophores was multiplied by two, namely from **5-M** to **5-G_0_**, then from **5-G_0_** to **5-G_1_**, and so on. No dendritic effect was observed in this case, since there was a linear correlation between the number of peripheral chromophores and the TPA cross-section values. The dendrimer **6-G_1_**, which has the same type of branches substituted by photochromic motifs, was built from a trifunctional core and was shown to exhibit a modest TPA efficiency with a *σ*(TPA) = 27 GM [29].

**Figure 4:**
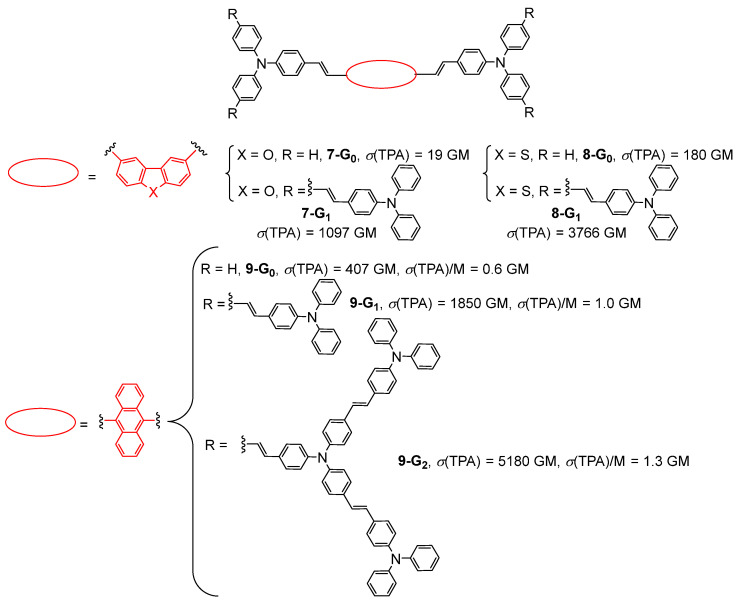
Bis-(diphenylamino)stilbene-based dendrimers with various cores.

The effect of using dibenzofurane or dibenzothiophene as the cores of first- and second-generation bis-(diphenylamino)stilbene-based dendrimers **7-G_n_** and **8-G_n_** (n = 0, 1) on the TPA properties was reported in 2009 (Figure 4) [30,31]. Later, the properties of the same kind of bis-(diphenylamino)stilbene-based dendrimers **9-G_n_** (n = 0–2) with an anthracene core were investigated up to the second generation (Figure 4) [32]. These studies showed that the effect of the modification of the core of the dendrimer on its TPA cross-section is more important than the effect of the increase in generation. In particular, the TPA cross-section of **9-G_0_** (407 GM) with an anthracene core is more than 20 times higher than that of the benzofurane-cored analog **7-G_0_** (19 GM). In the case of the anthracene dendrimer **9-G_n_**, a dendritic effect was observed upon increasing the generation, as evidenced by the values of TPA cross-sections divided by the molecular weights.

**Figure 5:**
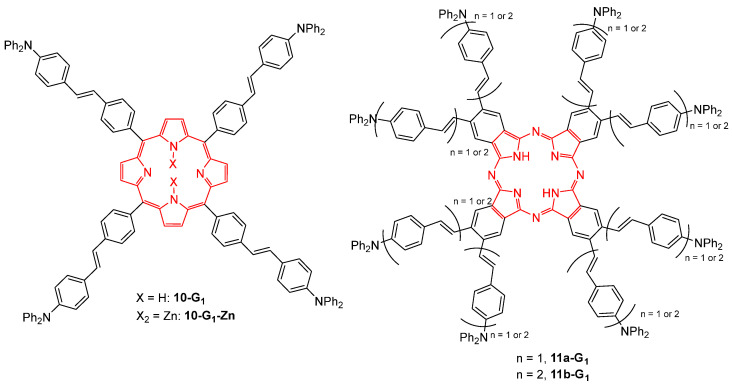
Dendritic TPA chromophores with tetrapyrrolic cores.

Four first-generation dendrimers with a tetrapyrrolic core, either a porphyrin core bearing four diphenylaminostilbene branches **10-G_1_** and the metalated analog **10-G_1_-Zn**, or a phthalocyanine core and eight branches **11a,b-G_1_**, were investigated for their TPA properties (Figure 5) [33]. While the phthalocyanine dendrimers **11a,b-G_1_** showed almost no cooperative effect, their TPA efficiency corresponding to that of their branches, the porphyrin counterparts **10-G_1_** and **10-G_1_-Zn** were found to exhibit a very strong cooperative effect. For example, the TPA cross-section of **10-G_1_** is nine times larger than the sum of those of its constituents. This difference between the two cores was explained by the less efficient conjugation along the tetrapyrrolic macrocycle in the case of the phthalocyanine core.

**Figure 6:**
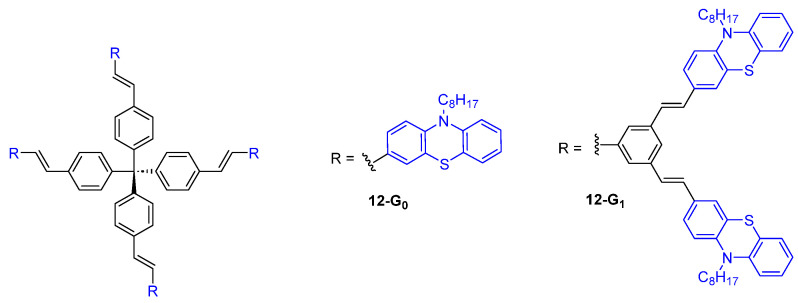
Dendrimers with a tetraphenylmethane core and phenothiazine substituents.

Dendrimers with a tetraphenylmethane core and phenothiazine fluorophores at the periphery were also considered as TPA chromophores and were shown to exhibit a moderate TPA cross-section of 89 GM for **12-G_0_** and 343 GM for **12-G_1_** (Figure 6) [34].

**Figure 7:**
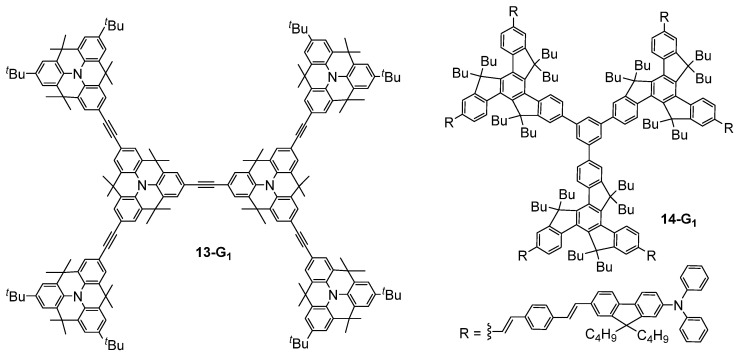
Structure of dendritic TPA chromophores with enhanced rigidity.

The structural modification of the bis-(diphenylamino)stilbene dendrimers was also envisaged by replacing the double bonds of **4-G_n_** (Figure 2) with triple bonds [35], thus rigidifying the structure, or by replacing the nitrogen atoms of **4-G_n_** with 1,3,5-substituted benzene rings [36], but without a significative effect on the obtained TPA cross-section values. Related triphenylamine-based dendrimers **13-G_n_** in which the triphenylamine moieties were linked by methylene bridges, thus rigidifying the structure a lot as compared to classical triphenylamine-based dendrimers such as **4-G_n_** (Figure 2), were also considered as TPA chromophores [37]. This enhancement of the rigidity and planarity of the molecules was shown to improve their TPA efficiency, reaching values up to 6100 GM for a first-generation dendrimer **13-G_1_** (Figure 7). Another rigidified small dendrimer **14-G_1_** with six chromophores at the periphery was also described as presenting quite a high TPA cross-section of 1400 GM [38].

**Figure 8:**
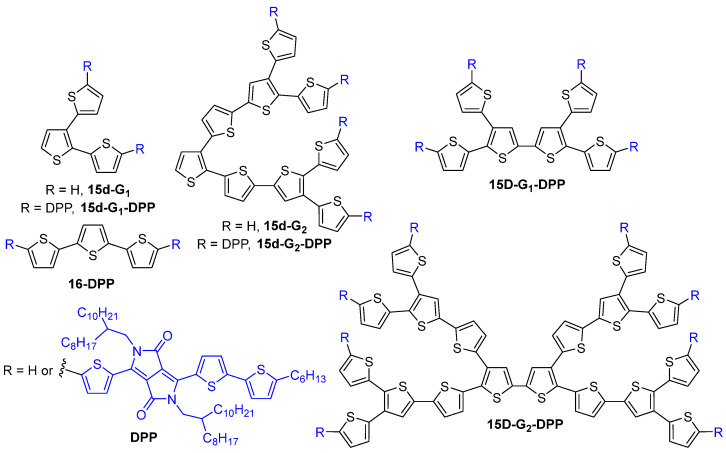
All-thiophene dendrons and dendrimers.

The TPA properties of all-thiophene dendrons **15d-G_1–4_** up to the fourth generation were investigated and a linear increase of the TPA cross-section values was observed upon increasing the dendrimer generation (Figure 8) [39]. All-thiophene dendrimers were also studied by entangled two-photon absorption [40,41,42], entangled photons being pairs of photons with a high degree of temporal and spatial correlation. In the entangled two-photon absorption process, the transition between the ground state and the two-photon excited state is accomplished in a single step by a pair of entangled photons [43]. The entangled TPA values of these dendrimers were found to follow the same trends of the values measured by the classical Two-Photon Excited Fluorescence (TPEF) method, although 10 orders of magnitude fewer photons are used. 

The same kind of all-thiophene dendrons of the first, second and third generations **15d-G_1–3_-DPP** and the corresponding first- and second-generation dendrimers **15D-G_1–2_-DPP** with diketopyrrolopyrrole groups at the surface were also described as efficient TPA chromophores (Figure 8) [44]. Their TPA efficiency was compared to that of a linear derivative **16-DPP**. As compared to the linear molecules, the dendrons and dendrimers exhibited enhanced TPA cross-section values that could be assigned to the 3D structure of the dendritic compounds. Indeed, the value obtained for **15d-G_1_-DPP** was 215% higher than the one obtained for **16-DPP**, which has the same molecular weight but a linear instead of a branched structure. Increasing the number of branches, going from the first-generation dendron **15d-G_1_-DPP** to the first-generation dendrimer **15D-G_1_-DPP**, also increased the TPA cross-section value from 2499 GM to 6984 GM. However, it was shown that increasing the generation did not induce a further enhancement of the TPA efficiency, probably because of a less efficient orbital overlap due to steric hindrance.

**Figure 9:**
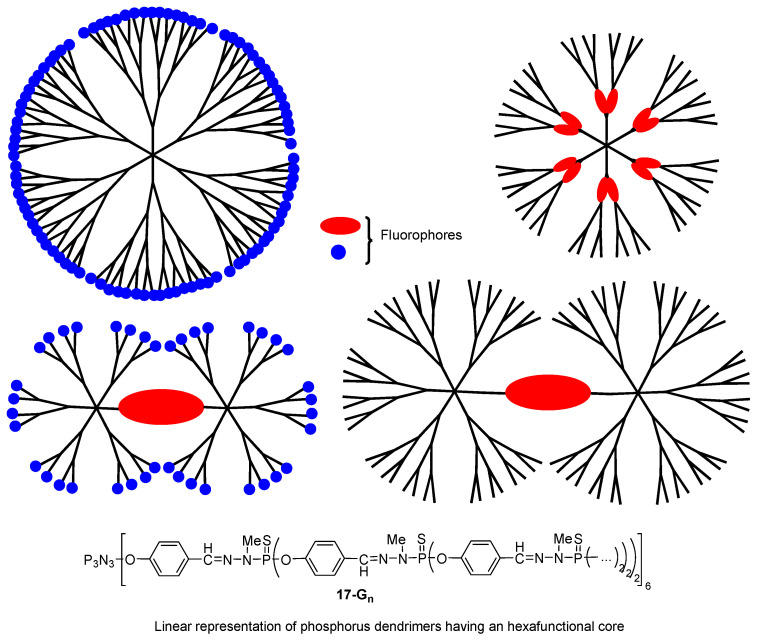
Schematic representations of the different types of phosphorous dendrimers embedding fluorophores in their structure.

The phosphorus dendrimers **17-G_n_**, developed by Anne-Marie Caminade and Jean-Pierre Majoral for more than 25 years [45], were also envisaged as TPA chromophores. More precisely, this family of arborescent molecules was proposed to be used as organic “nanodots”, alternatives to inorganic quantum dots that were shown to exhibit a very high TPA efficiency [46]. Various chromophores such as fluorene, stilbazole, and Nile Red derivatives were introduced either at the surface [47,48,49], at the core [50], or inside the branches of phosphorus dendrimers [51]. Phosphorus dendrimers with chromophore units both at the core and at their periphery were also envisaged (Figure 9) [52]. All these data were described in a recent review [53]. Some of these dendritic “nanodots” were shown to exhibit very high TPA cross-section values, outperforming those of quantum dots. The highest TPA cross-section value of 55,900 GM was measured for a fourth-generation dendrimer with 96 fluorene-based fluorophores at the periphery [47].

The two-photon absorption ability of dendrimers has been largely studied, as illustrated above, but very often this property is targeted with a particular objective related to a prospect of application. Hereafter, an overview of the different types of applications that have been envisaged for dendritic TPA chromophores is presented.

## 3. Fluorescence Resonance Energy Transfer and Light-Harvesting Properties of Dendritic TPA Chromophores

Theoretical studies of the energy migration processes in dendrimers after two-photon excitation, and thus of their light-harvesting properties, were carried out with the objective of clarifying the different mechanisms of energy transfer that are involved in these multichromophoric molecules [54,55]. Experimentally, dendrimers of the type **5-G_n_** (Figure 3), with two or four donor TPA chromophores at the periphery playing the role of antennas and a Nile Red acceptor at the core, were studied for their light-harvesting properties [56,57]. The emission from the core of the dendritic system was shown in this work to be stronger than the emission of the Nile Red core alone, thanks to the much larger TPA cross-section of the donor chromophores present at the surface of the dendrimer and to the efficient Fluorescence Resonance Energy Transfer (FRET) from these peripheral antennas to the acceptor core.

**Figure 10:**
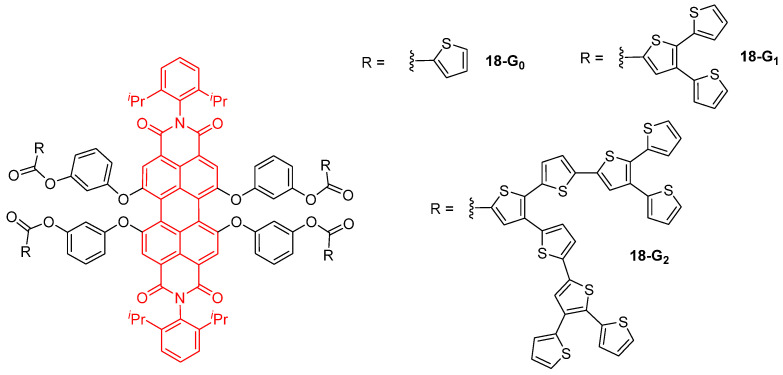
Light-harvesting properties of thiophene-based dendrons grafted onto a perylene bisimide core.

All-thiophene dendrons **15d-G_n_** (Figure 8) and thiophene-based dendrons grafted on a perylene bisimide core **18-G_n_** (Figure 10) were also studied for their light-harvesting properties [39,58]. In the all-thiophene dendrimers **15d-G_n_**, the presence of excited states delocalized over a large part of the dendrimer, and an ultrafast energy transfer along the branches were evidenced, thus confirming their efficient light-harvesting ability. The TPA properties of the perylene bisimide-cored thiophene dendrimers **18-G_n_** were investigated, showing that the first-generation derivative is more efficient than the second generation analog, because of the torsion of the perylene core caused by the increased hindrance brought by the dendrons. As in the all-thiophene series, an ultrafast energy transfer from the thiophene dendrons to the perylene core was observed, accompanied by an electron transfer inducing the formation of a perylene anion radical at about 700 nm and of a thiophene cation radical at about 500 nm. Because of the torsion of the core when grafted with second generation dendrons, and because of the stronger overlap of the dendrons due to their larger size, electrons are delocalized throughout the full molecule after excitation, thus increasing the number of pathways for electron transfer and inducing a faster energy transfer rate than in the smaller dendrimers.

**Figure 11:**
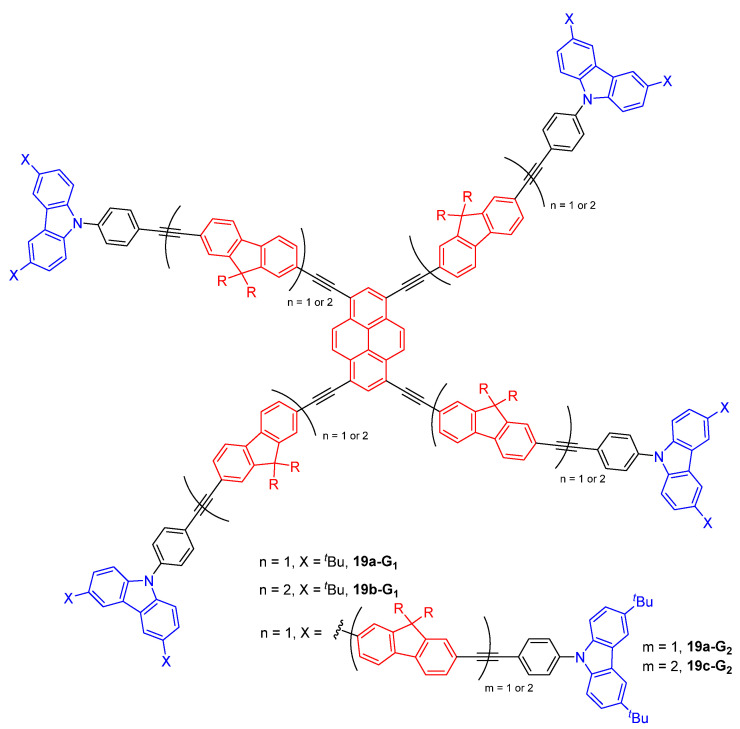
Dendrimers with a pyrene core and fluorene/carbazole branches as TPA chromophores with light-harvesting properties.

Dendrimers with a pyrene core and branches made of fluorene and carbazole motives connected to each other by acetylenic linkers were also synthesized and studied experimentally and theoretically for their TPA and light-harvesting properties (Figure 11) [59,60]. A TPA cross-section value up to 25,000 GM was measured for the larger dendrimer **19c-G_2_**, but this value is lower than that obtained for the smaller **19a-G_1_** if the values are given divided by the molecular weight. However, the addition of a second fluorene moiety in the branches going from **19a-G_2_** to **19c-G_2_** induces an important increase of the TPA efficiency (from 15,200 to 25,000 GM, from 2.45 to 3.15 GM/MW), showing that the constitution of the branches has a big impact on the cross-section values. An efficient intramolecular FRET from the branches to the core was also evidenced. The energy transfer efficiency was shown to be above 98% for the two first-generation dendrimers, and to slightly decrease to 97% and 95% for the second generation derivatives **19a-G_2_** and **19c-G_2_**, respectively. These energy transfers were described to result from through-space Förster energy transfer and not from through bonds energy transfer, because the delocalization in the branches is interrupted at the level of the carbazole motive. Theoretical calculations performed on this series of dendrimers showed that the existence of intermolecular couplings between the conjugated chromophores in the branches and the core significantly favors the TPA.

Bis-(diphenylamino)stilbene-based dendrimers with the same branches as **4-G_n_** (Figure 2) and a 1,3,5-substituted benzene core were also investigated for their TPA and light-harvesting properties [61]. The TPA cross-section (*σ*(TPA)/MW) of the second generation derivative was measured to be more than twice that of the first-generation analog, thus suggesting that there is a cooperative enhancement of the TPA and of the light-harvesting ability due to the increase of the dendrimer size and to inter-branch coupling.

## 4. TPA-Induced Photopolymerization with Dendrimers

**Figure 12:**
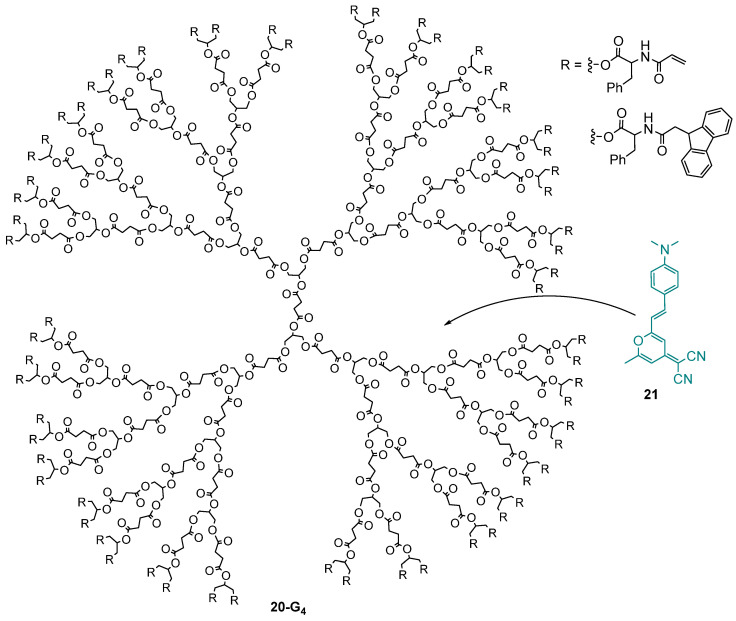
Dendrimer encapsulating TPA dyes for TPA-induced photopolymerization.

The use of the TPA process to induce photopolymerization is very attractive because it allows the fabrication of three-dimensional submicrometer structures. The introduction of the TPA chromophores into dendrimers limits their interaction and thus limits the fluorescence quenching. The dendrimer **20-G_4_** encapsulating the TPA chromophore **21** and bearing two kinds of peripheral groups (Figure 12) was used for the TPA-induced fabrication of microstructures with high spatial resolution [62,63,64]. The dendrimer **20-G_4_** was shown to be a good host for this TPA dye, and no leaching was observed thanks to the bulky groups at the surface of the dendrimer. Then, the dendrimer encapsulating the dyes was mixed with a prepolymer and a radical photoinitiator, and the polymerization was performed by laser irradiation at 780 nm. The use of dendrimers allowed for the introduction of a higher concentration of the TPA chromophore **21**, ca 4.2 wt% versus only 0.5 wt% without the dendrimer, and favored the photopolymerization while avoiding undesirable energy transfers between the photoinitiator and the chromophore.

**Figure 13:**
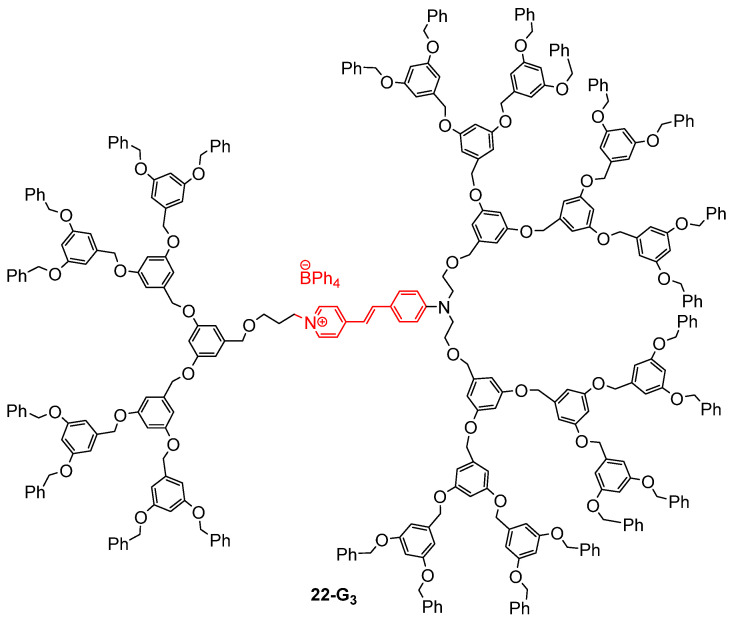
Fréchet-type dendrimer with a stilbazolium core for TPA-induced polymerization.

The dendrimer **22-G_3_** made of Fréchet-type dendrons attached on a stilbazolium salt acting as a TPA chromophore was also applied for TPA-induced photopolymerization (Figure 13) [65,66]. In this case, the TPA dye was not encapsulated in the cavities of the dendrimer, but located at its core, thus preventing the chromophores’ aggregation thanks to the steric hindrance brought by the surrounding dendrons. A total of 0.1% of this dendrimer was introduced as photosensitizer in a resin which was used for TPA-induced polymerization, thus allowing the fabrication of micro-patterns and of a three-dimensional object with a high spatial resolution.

First- to third-generation dendrimers of the type **9** (Figure 4), but with a naphthalene instead of an anthracene core, were also envisaged as photoinitiators for TPA-induced polymerization [67]. Small amounts of these dendrimers of various generations were introduced in resins and tested as initiators of polymerization and were all shown to be efficient. The authors also demonstrated that the light energy was absorbed by the triphenylamine peripheral groups of the first-generation dendrimer and then transferred to the core. They also showed that there was no intermolecular energy transfer between the dendrimer and the monomer, but that an electron transfer occurred, thus inducing the polymerization.

## 5. Optical Power Limitation with Dendrimers

Optical power-limiting materials are developed to protect devices or human eyes against laser light and require a high transmission of low-intensity light and a reduced transmission of high-intensity light. Among the mechanisms leading to optical limitation, one can find the TPA together with the excited-state absorption, and one way to increase the optical power-limitation efficiency is to control the intersystem crossing to triplet states and their lifetime. The bis(diphenylamino)stilbene-based dendrimers **2-G_0_** and **4-G_0_** (Figure 2) were investigated for their optical limitation properties and were shown to exhibit excited-state absorption, forming stable and strongly absorbing polarons and bipolarons [68,69]. Theoretical calculations were also performed on the dendrimer **2-G_0_** and on analogs in which the nitrogen atoms were replaced by boron or aluminum. These dendrimers were calculated to exhibit large TPA cross-sections and a good transparency, making them promising for optical limitation [26].

**Figure 14:**
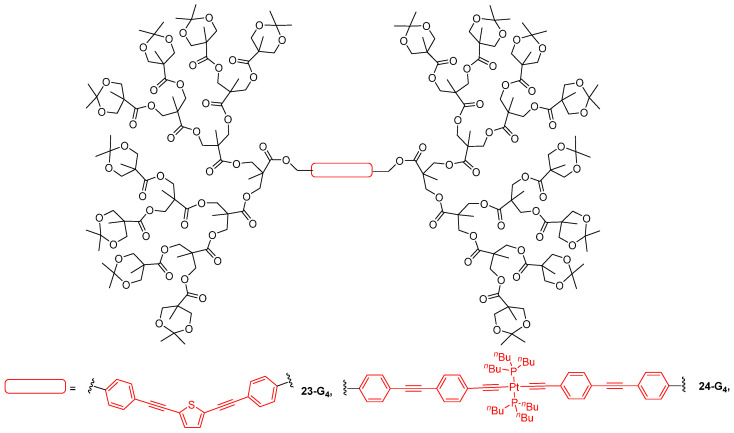
Fourth-generation dendrimers made of non-conjugated dendrons grafted onto TPA chromophores.

Non-conjugated first- to fourth-generation dendrons were attached onto two different conjugated cores, a bis-arylethynylthiophene and a platinum bis-acetylide, thus giving the dendrimers **23-G_1–4_** and **24-G_1–4_**, respectively, which were evaluated for both their TPA properties and optical power-limiting behavior (Figure 14). Here, the role of the dendrons was to protect the central TPA chromophores from aggregation, and to decrease the excited-state quenching, thus leading to better excited-state absorption and better optical power limitation. For the dendrimers **23-G_1–4_** with a thiophene core, the size of the dendrons was shown to have no influence on the optical properties, and their optical power-limitation efficiency was found to be lower than that of the core substituted by simple alkyl chains [70]. However, the dendrimers **24-G_1–4_** with a platinum acetylide core appeared to exhibit enhanced optical power-limitation properties as compared to the chromophore alone [71]. This improvement was suggested to originate from excited-state absorption and the longer lifetime of the excited triplet states occurring thanks to the protection brought by the dendrons. A similar effect was observed for the dendrimer **22-G_3_** (Figure 13), which was described as having a much higher optical power-limitation efficiency than the stilbazolium salt alone because of the site isolation effect provided by the bulky dendrons attached to the chromophore [66].

**Figure 15:**
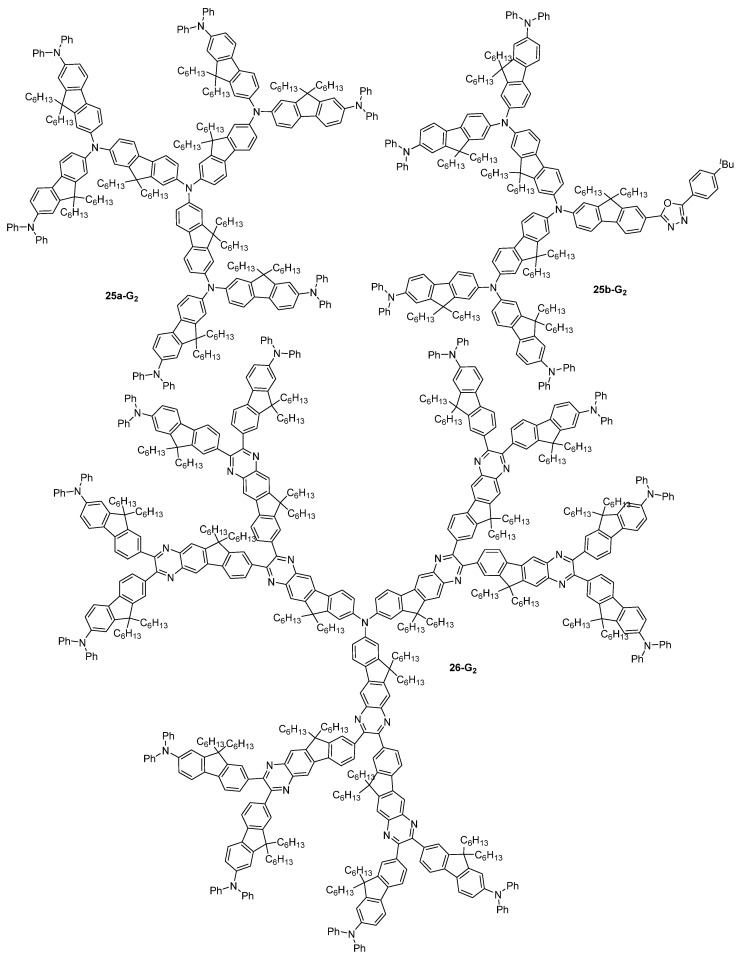
Dendrimers and dendrons based on trifluorenylamine motives **25a-G_2_** and **25b-G_2_**, and indenoquinoxaline motives **26-G_2_**.

Dendrimers of the first to third generations **25a-G_1–3_** made of donor trifluorenylamine motives and analogs **25b-G_1–3_** with an oxadiazole acceptor on one branch were also considered for TPA-induced optical power limitation (Figure 15). One oxadiazole group was introduced on one branch of **25b-G_1–3_** with the objective of exploring the influence on the TPA properties of this unique acceptor group, while the other branches of the molecules were composed of a variable number of donor groups. Indeed, it was proposed that a multi-polar character could modify the charge transfer in the molecules upon light excitation. These investigations showed that the increase in the number of donor branches upon increasing the dendrimer generation induced an increase in the TPA efficiency, and that the **25a-G_3_** displayed a better optical power limitation than the smaller analogs [72]. The corresponding symmetrical dendrimers **25b-G_1–3_** only made of donor fluorenylamine groups were shown to also be efficient TPA chromophores, the cross-section increasing with the increase of the size of the dendrimer [73]. These dendrimers also exhibited strong two-photon-assisted excited-state absorption, and an effective optical power-limiting behavior. The related dendrimer **26-G_2_**, in which indenoquinoxaline motives were introduced in the branches, was also shown to exhibit a high TPA cross-section in the near-IR region and an efficient optical power-limitation behavior [74].

## 6. Dendrimers for Cell Imaging

**Figure 16:**
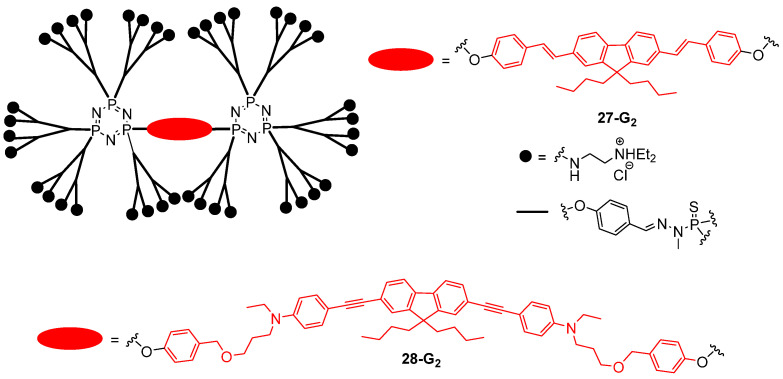
Water-soluble phosphorous dendrimer with a chromophore at the core and ammoniums at the periphery.

The use of two-photon excited fluorescence for cell imaging is attractive because it allows reaching a high spatial resolution in three dimensions and because it allows imaging at high depth in tissues with reduced photodamages, thanks to the use of near-infrared excitation wavelengths. It requires the design of water-soluble chromophores with both a high fluorescence quantum yield and a high TPA cross-section upon irradiation at wavelengths of interest for bioimaging, typically between 700 and 1000 nm. To fulfill all these requirements, the use of dendrimers was envisaged. In 2006, the synthesis of the polyphosphorhydrazone dendrimers **27-G_1–3_** with a lipophilic TPA chromophore at the core and cationic groups ensuring water solubility at the periphery was described [75]. These dendrimers were shown to exhibit a significant TPA cross-section value in water, the dendritic branches surrounding the chromophore preventing its aggregation and thus fluorescence quenching in water. The second generation dendrimer **27-G_2_** (Figure 16) was used for the two-photon imaging of the vascular network of the rat olfactory bulb without any observed toxicity, thus demonstrating the relevance of this approach. The dendrimer **28-G_2_** with a different chromophore at the core was also used as a contrast agent for the in vivo imaging of the blood vessels of the tail of the Xenopus tadpole by TPEF [76].

**Figure 17:**
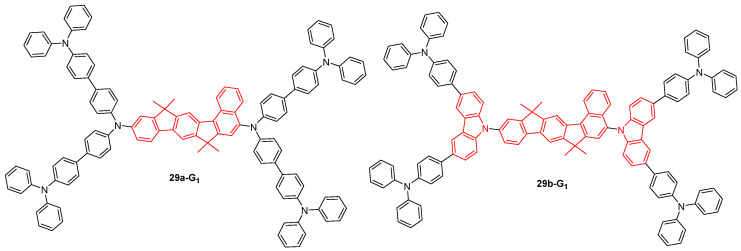
Naphthalene indenofluorene-based dendrimers used for two-photon excited fluorescence imaging.

First-generation dendrimers **29a-G_1_** and **29b-G_1_** based on naphthalene indenofluorene motives were also used for two-photon fluorescence imaging (Figure 17) [77]. These lipophilic molecules showed high TPA cross-section values and an intense two-photon excited fluorescence in organic solvents. Organic nanoparticles were prepared by encapsulation of these dendrimers in a biocompatible polymer matrix and suspended in aqueous media. These nanoparticles still exhibited TPA properties in water, in particular those prepared from **29a-G_1_**. They were shown to be non-cytotoxic and were applied for the two-photon fluorescence imaging of hepatic tissues and blood vessels.

## 7. Dendritic TPA Chromophores for the Generation of Singlet Oxygen

**Figure 18:**
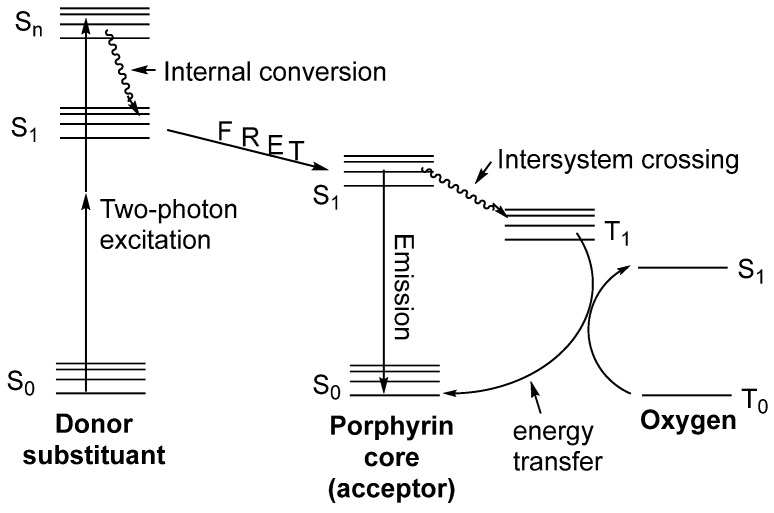
Jablonski diagram illustrating the processes involved in the generation of singlet oxygen by two-photon excitation of porphyrin-cored dendrimers. This figure was inspired by Ref. [80].

Dendrimers with a porphyrin core were largely investigated for the generation of cytotoxic singlet oxygen upon two-photon excitation. Indeed, porphyrins and related tetrapyrrolic molecules are TPA chromophores, making them attractive for biological applications including two-photon photodynamic therapy, since TPA photosensitizers allow for using wavelengths in the near-IR region where living tissue is more transparent [78]. Arborescent substituents on porphyrins are of interest because their peripheral groups can serve as antennas and transfer their energy by FRET to the tetrapyrrolic core, thus increasing their TPA efficiency, but also because they can be used to provide water solubility to the whole molecule, making it suitable for biological applications. The mechanism leading to the production of singlet oxygen by two-photon excitation of dendrimers with a porphyrin core is shown in Figure 18. After absorption of two photons by the TPA chromophores at the surface of the dendrimers, the energy is transferred by FRET to the porphyrin core, which is then in its singlet excited state S_1_. Then, intersystem crossing induces the population of the triplet state T_1_ of the porphyrin, which is deactivated by collision with triplet oxygen, thus producing singlet oxygen. 

**Figure 19:**
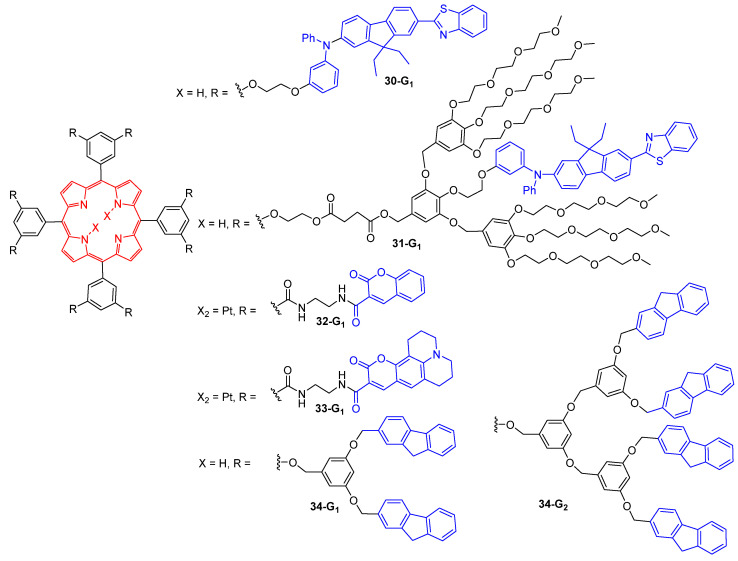
Dendrimers with a porphyrin core and various TPA chromophores at the surface.

The first example of a porphyrin-cored dendrimer which was used for TPA-induced singlet oxygen generation was reported in 2003 for the dendrimer **10-G_1_** (Figure 5) [24]. Then, the introduction of various donor TPA chromophores such as AF-343, coumarin derivatives or fluorene groups at the periphery of dendrimers with an acceptor porphyrin core and various types of branches was largely investigated. In 2004, the first-generation dendrimer **30-G_1_** bearing eight AF-343 chromophores was shown to have a larger TPA cross-section than the non-dendritic porphyrin, allowing for a more efficient singlet oxygen generation under irradiation at 780 nm (Figure 19) [79]. Introduction of polyethylene glycol (PEG) groups on the dendrimer **31-G_1_** bearing the same number of AF-343 chromophores was then envisaged to make them water soluble [80]. The authors demonstrated that the singlet oxygen generation process starts with the absorption of two photons by the peripheral chromophores. Then, a fluorescence resonance energy transfer from the donor chromophore to the acceptor porphyrin occurs, inducing the indirect excitation of the latter, which finally produces singlet oxygen. The singlet oxygen generation with dendrimers with a metalloporphyrin core was also investigated with the derivatives **32-G_1_** and **33-G_1_**, with donor coumarin substituents at the periphery. In this case also, the introduction of peripheral groups bringing water solubility to these dendrimers was envisaged [81].

Dendrimers **34-G_1_** and **34-G_2_** with fluorenyl peripheral groups were reported to exhibit enhanced TPA cross-section as compared to the model tetraphenylporphyrin (TPP), and to generate singlet oxygen with quantum yields slightly higher than that obtained with TPP [82].

**Figure 20:**
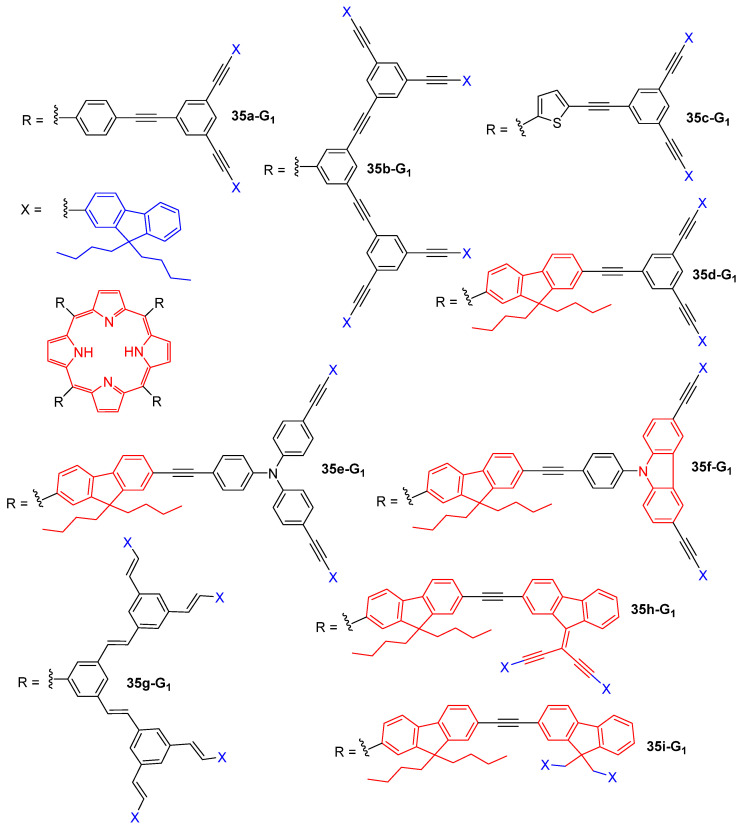
Porphyrin-cored dendrimers with peripheral fluorene groups and various types of branches.

Porphyrin-cored dendrimers with fluorene surface groups but a different type of branches were also largely studied. Indeed, while **34-G_1_** and **34-G_2_** are made of non-conjugated polyether dendrons, dendrimers **35a-G_1_** and **35b-G_1_** with phenylacetylene branches are π-conjugated (Figure 20). Dendrimers **35a-G_1_** and **35b-G_1_**, which have a more rigid and electron-rich backbone, were shown to exhibit significantly improved TPA efficiencies and singlet oxygen generation quantum yields as compared to **34-G_1_** and **34-G_2_**. A very efficient FRET from the fluorene peripheral groups to the central porphyrin was also evidenced for this series of conjugated dendrimers. It was also demonstrated that when the dendrons were attached at the *para*-position of the TPP (**35a-G_1_**), both their TPA and singlet oxygen generation properties were enhanced as compared to the dendrimers **35b-G_1_**, which have the dendrons at the two *meta*-positions of the central TPP [83,84]. The dendrimer **35c-G_1_** devised from the tetrathienylporphyrin was shown to exhibit enhanced TPA and oxygen generation efficiencies as compared to **35a-G_1_** devised from TPP [85]. In the same way, dendrimers **35d-G_1_** and **35d-G_2_** with a tetrafluorenylporphyrin core were reported to display increased TPA cross-sections as compared to TPP-based analogs **35a-G_1_**, and similar quantum yields of oxygen generation [86]. The TPA and oxygen generation properties of dendrimers **35e-G_1_** and **35f-G_2_** obtained by the introduction of triphenylamine of phenylcarbazole motifs, respectively, in the branches were also investigated, showing that the triphenylamine groups had a positive effect on these properties while the phenylcarbazole had a negative effect. The corresponding dendrimers with a Zn-metalated porphyrin core also were studied and were found to be less efficient for both properties [87]. The replacement of the triple bonds of **35a-G_1_** with double bonds in **35g-G_1_** allowed for the enhancement of both the TPA cross-section values and the singlet oxygen generation efficiency, and the zinc complexation of the porphyrin core was shown not to affect these properties [86].

The insertion of more fluorenyl groups into the branches of **35d-G_1_**, either in a conjugated or non-conjugated way in **35h-G_1_** or **35i-G_1_**, respectively, was also considered. The conjugated representative **35h-G_1_** was shown to be a particularly efficient TPA chromophore and to exhibit a high two-photon-induced singlet oxygen generation efficiency, the fluorene connectors allowing the improvement of the electronic communication between the branches and the porphyrin core, as compared to the 1,3,5-phenylene connectors of **35d-G_1_** [88].

Dendrimers of the zeroth, first, and second generations, with a zinc-phthalocyanine core instead of a porphyrin and the same branches as **35a-G_1_**, were also studied in order to evaluate the effect of changing the type of tetrapyrrolic core. The TPA efficiency of these chromophores was shown to increase upon increasing the size of the dendrimer, but if the TPA cross-sections were expressed related to the number of active π-electrons, the smaller dendrimers were found to be more efficient than the larger ones [89]. However, the performances of these dendrimers are comparable with those of other phthalocyanine-based systems already applied as one- or two-photon photosensitizers used in cancer therapy [90].

## 8. Dendritic TPA Chromophores for Photodynamic Therapy

**Figure 21:**
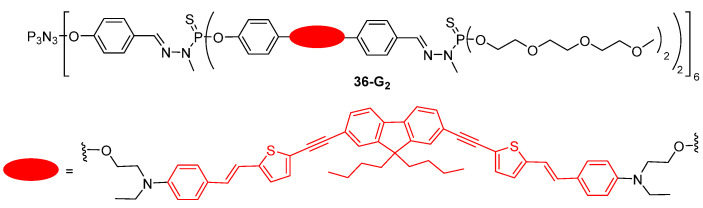
Phosphorus dendrimer with chromophores inside the branches for photodynamic therapy and cell imaging.

Photodynamic therapy, based on the use of photosensitizers that can generate reactive oxygen species upon irradiation, is one of the therapeutic options that can be used for the treatment of cancers, since the produced radicals induce the death of the treated cells without affecting the neighboring healthy tissues. The use of TPA activation for photodynamic therapy is an attractive approach because it allows an increase in the light penetration through the use of near-infrared lasers and a decrease in the damages induced to neighboring tissues. As described in the Section 7, a lot of dendrimers, in particular those with a tetrapyrrolic core, were shown to be able to produce singlet oxygen upon two-photon excitation. If the chromophore is fluorescent, then, in vitro and in vivo monitoring by fluorescence imaging is possible, thus allowing for theranostic applications. In this context, the second generation polyphosphorhydrazone dendrimer **36-G_2_** with TPA chromophores inside the branches and water-solubilizing PEG groups at the periphery was synthesized (Figure 21) [91]. Thanks to fluorescence imaging experiments, the dendrimer was shown to be able to penetrate inside cancer cells and to concentrate into lysosomes, without observed toxicity in the absence of irradiation. In vitro photodynamic therapy experiments were performed on breast cancer cells, and a toxicity was observed, possibly originating from singlet oxygen production induced by two-photon excitation of the chromophore-containing dendrimer.

**Figure 22:**
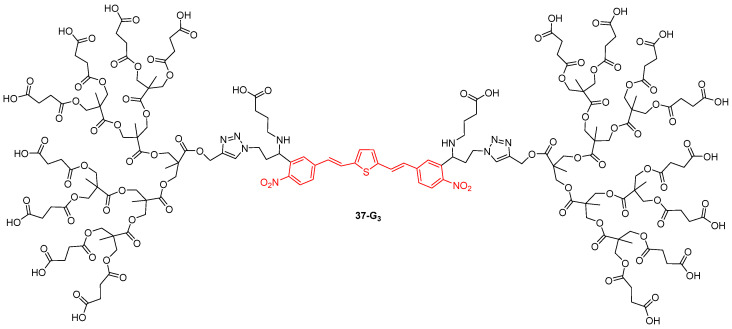
Dendrimer used for the TPA-induced photorelease of a neurotransmitter in living neurons.

The water-soluble dendrimer **37-G_3_** with an extended π-electron dinitrobisstyrylthiophene system, coupled with the γ-aminobutyric acid neurotransmitter at the core, was synthesized with the view to use it for the TPA-induced photorelease of the neurotransmitter (Figure 22) [92]. This dendrimer was shown to be non-toxic towards living neurons, while the core without the grafted dendrons is toxic. The uncaging of the neurotransmitter from the dendrimer by two-photon excitation could be demonstrated to be rapid and efficient, with a high spatial resolution.

## 9. Conclusions

**Figure 23:**
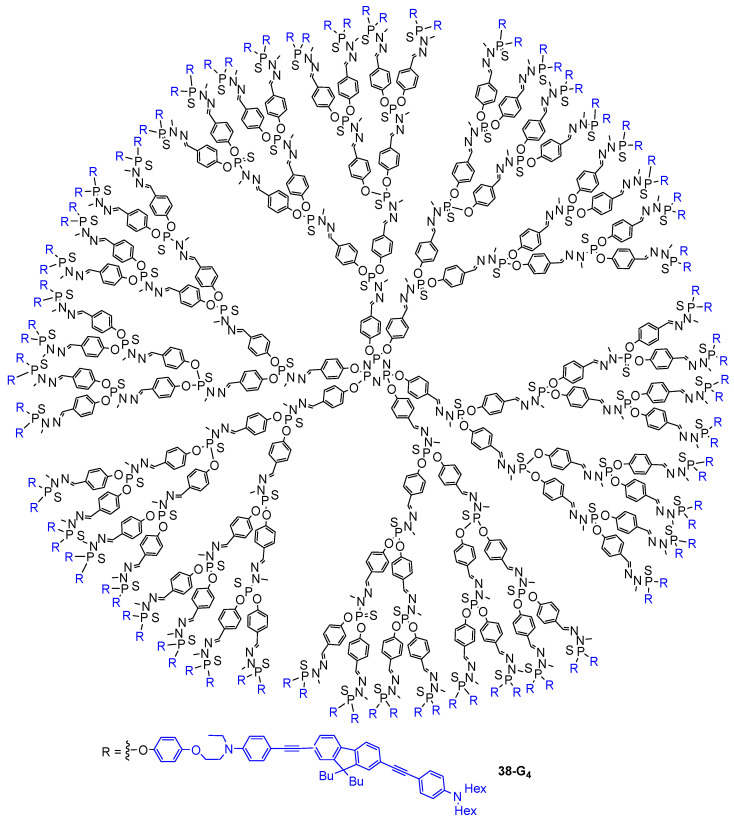
Polyphosphorhydrazone dendrimer with fluorophores at the periphery.

An overview of the dendrimers exhibiting two-photon absorption properties was described in this review. The possible applications related to the two-photon absorption process were shown to be varied and to open up prospects in many fields from materials to biology. This review covers the use of dendritic TPA chromophores for light harvesting, photopolymerization, optical power limitation, cell imaging, singlet oxygen generation, and photodynamic therapy. These aspects were the most surveyed in the literature, but other applications of dendrimers containing TPA chromophores were also more scarcely described. For example, fifth-generation PAMAM dendrimers stabilizing gold nanoparticles were shown to be able to stabilize charged metal clusters generated by multi-photon-induced fragmentation of the nanoparticles under irradiation [93]. Another example concerns the use of the fourth-generation polyphosphorhydrazone dendrimer **38-G_4_** with 96 electron-rich fluorophores at the periphery for sensing electron-deficient nitrated organic explosives, and in particular trinitrotoluene (TNT), by fluorescence quenching after two-photon excitation (Figure 23). The TNT molecule is detected by the fluorescence quenching induced by redox sensing, i.e., by its interaction with the electron-rich substituents at the periphery of the dendrimer [94]. There is probably still a lot to discover regarding the use of dendrimers for two-photon absorption-related applications, the possibilities of combining these tridimensional arborescent molecules with TPA chromophores being almost infinite.

It has been shown that very high TPA cross-section values can be reached with dendrimers, but in some cases, in particular when no dendritic effect is observed, the interest of using such large and expensive molecules can appear unnecessary. Nevertheless, the use of dendrimers remains attractive for bringing additional properties to the TPA chromophores, such as water solubility or protection against fluorescence quenching by isolation of the chromophore at the core or inside the dendrimer. Furthermore, high energy transfer efficiencies have been observed in dendrimers thanks to their well-defined arborescent structure, making them promising for uses in optoelectronic devices. Although the use of dendrimers presents several advantages due to their particular structure, the development of easy and fast synthetic approaches to prepare them is important to make them competitive against polymer counterparts.
